# Exploring β3-Adrenergic Receptor, HIF-1α, and CD31 Interplay in the Microenvironment of Atypical Melanocytic Lesions

**DOI:** 10.3390/dermatopathology13030031

**Published:** 2026-07-03

**Authors:** Eugenia Belcastro, Giuseppe Nicolò Fanelli, Cristian Fidanzi, Desirèe Fischetti, Riccardo Morganti, Katia De Ieso, Luca Filippi, Antonio Giuseppe Naccarato, Marco Romanelli, Cristian Scatena, Agata Janowska

**Affiliations:** 1 Department of Translational Research and New Technologies in Medicine and Surgery, University of Pisa, 56126 Pisa, Italy; 2 Division of Pathology, Department of Translational Research and New Technologies in Medicine and Surgery, University of Pisa, 56126 Pisa, Italy or gif4002@med.cornell.edu (G.N.F.); giuseppe.naccarato@unipi.it (A.G.N.); cristian.scatena@unipi.it (C.S.); 3 Department of Oncology, Pisa University Hospital, 56126 Pisa, Italy; ka.deieso@ao-pisa.toscana.it; 4 Department of Pathology and Laboratory Medicine, Weill Cornell Medicine, New York, NY 10021, USA; 5 Unit of Dermatology, Pisa University Hospital, 56126 Pisa, Italy; cri.fidanzi@outlook.it (C.F.); d.fischetti@studenti.unipi.it (D.F.); marco.romanelli@unipi.it (M.R.); agata.janowska@unipi.it (A.J.); 6 Melanoma and Skin Cancer Unit AVNO (Area Vasta Nord Ovest) Tuscany, Carrara Hospital, 54033 Carrara, Italy; 7 Department of Clinical and Experimental Medicine, University of Pisa, 56126 Pisa, Italy; luca.filippi@meyer.it; 8 Statistics Unit, Pisa University Hospital, 56126 Pisa, Italy; r.morganti@ao-pisa.toscana.it; 9 Neonatal Intensive Care Unit, Meyer Children’s Hospital IRCCS, 50139 Florence, Italy

**Keywords:** melanoma, tumor microenvironment, β3-adrenoceptors, hypoxia-inducible factor 1-alpha, immunohistochemistry

## Abstract

**Background:** Melanoma incidence is rising rapidly worldwide and stands out due to its high lethality. Despite advances in clinical treatment and in understanding melanoma-sensitive genes and molecular pathogenesis, a specific area of ongoing research is the connection between stress-related β-adrenergic receptors (ARs), hypoxia, and neovascularization in melanoma tumor progression. This exploratory study aimed to investigate the expression of β3-AR, HIF-1α, and CD31 in several cellular subsets of atypical melanocytic lesions and their interplay in promoting melanoma malignancy. **Methods:** Twenty-seven patients with melanocytic lesions at different stages that were surgically removed were retrospectively selected; clinical-pathological and dermoscopic data were collected. **Results:** Immunohistochemical and digital evaluation revealed a significant upregulation of β3-AR in malignant melanoma melanocytes and in macrophages from invasive >pT1a melanomas compared to dysplastic nevi. Increased HIF-1α expression in malignant melanocytes and CD31 expression levels in >pT1a melanomas were observed. Ulcerated lesions exhibited a higher percentage of β3-AR, HIF-1α, and CD31 expression. Pearson correlation analysis revealed positive associations among these markers in human malignant melanoma, suggesting a potential relationship between adrenergic signaling, hypoxia, and tumor vascularization. **Conclusions:** These exploratory findings suggest that β3-AR, HIF-1α, and CD31 may represent interconnected components of the melanoma microenvironment. Unraveling these interactions in larger, independent cohorts and functional studies may provide additional insights into melanoma biology and help define their potential translational relevance.

## Simple Summary

Melanoma is one of the most aggressive forms of skin cancer and identifying mechanisms that promote its progression remains a major challenge. In this exploratory study, we investigated β3-adrenergic receptor (β3-AR), hypoxia-inducible factor 1-alpha (HIF-1α), and the vascular marker CD31 in atypical melanocytic lesions ranging from dysplastic nevi to invasive melanomas. Immunohistochemical analysis showed increased β3-AR expression in melanoma cells and macrophages from more advanced lesions, together with higher HIF-1α and CD31 levels in invasive melanomas. Ulcerated lesions also displayed greater expression of these markers. Correlation analyses revealed significant positive associations among β3-AR, HIF-1α, and CD31, supporting a link between adrenergic signaling, hypoxia, and tumor vascularization. Although the study does not establish causal mechanisms, these findings suggest that these pathways may interact within the melanoma microenvironment and may be associated with tumor aggressiveness. Further studies are needed to clarify their biological and potential clinical relevance.

## 1. Introduction

Melanoma is one of the most aggressive and therapy-resistant skin cancers, and causes the majority of skin cancer-related deaths [1]. The worldwide incidence of melanoma has increased, and early diagnosis is crucial for a favorable prognosis [2]. Clinical and dermoscopic examinations are the standard diagnostic procedures, with dermoscopy enhancing diagnostic accuracy. Typical dermoscopic features of melanoma include multicomponent patterns with atypical features, such as an atypical network, irregular vessels, a blue-white veil, striae, pseudopodia, and milky-red areas. These features have been associated with invasive melanoma and increased angiogenic activity, and dermoscopy may reflect underlying biological processes within the tumor microenvironment [3]. Since hypoxia-driven angiogenesis is a key mechanism in melanoma progression, these dermoscopic patterns may reflect microenvironmental remodeling processes linked to hypoxia and stress-related signaling pathways [4]. From this perspective, dermoscopy may serve as a non-invasive tool for detecting molecular-level biological alterations [5]. Despite major advances in molecular tumor profiling that enable large-scale genomic analyses [6], the “gold standard” for melanoma diagnosis remains histopathology, often supported by immunohistochemistry and clinical correlation [7]. Dermoscopic and histological correlations have been extensively described in the literature [8].

Although advanced melanoma patients can benefit from immunotherapy and targeted therapy [9], the onset of resistance and severe side effects limits the percentage of patients with long-lasting responses. Indeed, this suggests that much remains to be investigated regarding the multi-step process of tumor metastasis, as well as the identification of novel therapeutic targets. Several studies have shown that the spread of melanoma results from genetic mutations and alterations in the tumor microenvironment, characterized by the overexpression of proteins that promote tumor invasion and infiltration [10]. In the search for possible novel targets to be used in treatment strategies to counteract melanoma growth, particular attention has been paid to catecholamines in tumor cells, mainly associated with β-adrenoceptors (β-ARs), a family composed of three members (β1, β2, and β3), which act through distinct and overlapping downstream pathways [11].

Primary melanoma cells express β1- and β2-ARs, with β2-ARs up-regulated in metastatic melanoma and strongly correlated with malignancy [12]. These receptors are strongly expressed and functionally active in a wide range of tumor-associated cells, including cancer-associated fibroblasts, macrophages, and endothelial cells, thereby modulating the tumor microenvironment [13]. Catecholamines recruit and stimulate endothelial cells to proliferate and form tube-like structures [14] and promote macrophage polarization toward the tumor-permissive M2 phenotype [14], facilitating melanoma progression.

Recently, β3-ARs have been shown to be expressed by murine melanoma B16F10 cells and by endothelial cells of the tumor vasculature [15,16], suggesting a broader role for β-AR signaling in modulating melanoma biology [15]. In human melanoma cells, β3-ARs may induce the expression of pro-angiogenic factors, including VEGFs and some pro-inflammatory cytokines (e.g., interleukin-6 (IL-6), IL-8, TNFα) [12,14,17], and activate the endothelial NOS (eNOS/NOS-3) pathway [18], indicating that catecholamines may influence tumor progression by regulating angiogenesis and tumor metastatization in the hypoxic environment [12,17]. Hypoxia activates hypoxia-inducible factor 1-alpha (HIF-1α), which promotes tumor growth by regulating the expression of genes involved in angiogenesis, metabolism, cell proliferation, and metastasis [5,19,20]. HIF-1 has also been implicated in cutaneous melanoma heterogeneity and in triggering metastatic progression by driving a switch from a proliferative to an invasive phenotype [5]. Indeed, under hypoxic conditions, melanoma cells upregulate β3-AR expression in parallel with VEGF production, representing an adaptive response to low oxygen tension. Moreover, β3-ARs facilitate the recruitment and differentiation of circulating stromal cell precursors at tumor sites, thereby further promoting tumor inflammation, angiogenesis, and melanoma progression [21].

Furthermore, tumor growth requires a sufficient blood supply, and microvascular density (MVD) remains the key morphological indicator of neovascularization and a prognostic marker in cutaneous melanomas [22]. Different pan-endothelial cell markers have been used to estimate MVD, with CD31 and CD34 among the most widely accepted markers for characterizing and studying tumor cell vasculogenic mimicry and malignant neoangiogenesis [22].

Against this background, the present study aimed to evaluate the immunohistochemical expression of β3-AR, HIF-1α, and CD31 in atypical melanocytic lesions and to investigate their possible interplay in promoting melanoma malignancy.

## 2. Materials and Methods

### 2.1. Case Selection

A total of 27 patients who had previously undergone removal of atypical melanocytic lesions at the Department of Dermatology were retrospectively enrolled, and their formalin-fixed, paraffin-embedded (FFPE) samples were selected from the archives of the Pathology Unit of the University Hospital of Pisa (from 2020 to 2022). According to histological results and AJCC 8th Edition Guidelines [23], the patients were categorized into four groups: dysplastic nevi (including high-grade dysplastic nevi) (*n* = 7), melanomas in situ (*n* = 6), pT1a melanomas (*n* = 8), and >pT1a melanomas (including melanomas pT2a, pT3a, pT3b, and pT4a) (*n* = 6). All histopathological diagnoses were confirmed by an expert dermatopathologist (C.S.) before study inclusion. The study was conducted according to the guidelines of the Declaration of Helsinki and approved by the Ethical Committee for the testing and evaluation of clinical study protocols in the Northwestern Tuscany Area (CEAVNO) (protocol code: MIRACLE; approval date: 20 May 2022; chairperson: Dr. Diego Carignani). Written informed consent was obtained from each participant. Clinical–pathological and dermoscopic data, such as age, gender, anatomical localization, dermoscopic features (e.g., presence of ulceration, blue-white veil, regression, and atypical network), histopathological ulceration, tumor-infiltrating lymphocytes (TILs), Breslow thickness, and stage, were annotated. Dermoscopic features were collected as descriptive clinical variables to complement the clinicopathological characterization of the lesions and were included as adjunct parameters in the exploratory analysis.

### 2.2. Immunohistochemistry (IHC)

Sections of 4 μm thickness were cut from FFPE tissue blocks. Slides were deparaffinized using EZ prep solution (Ventana Medical Systems, Inc., Tucson, AZ, USA) at 72–75 °C. Immunohistochemistry was performed on a VENTANA BenchMark Ultra automated staining platform (Roche Diagnostics, Basel, Switzerland), and staining was developed with 3-amino-9-ethylcarbazole (AEC; Ventana AEC Detection Kit, Basel, Switzerland). Epitope retrieval for beta-3 adrenergic receptor (β3-AR) was performed using citrate buffer and Cell Conditioning 2 solution (Ventana Medical Systems, Inc., Basel, Switzerland, Cat# 05279798001) for 44 min. Epitope retrieval for HIF-1 alpha (HIF-1 α) and CD31 was performed using EDTA buffer and Cell Conditioning 1 solution (Ventana Medical Systems, Inc., Basel, Switzerland, Cat# 950–500) for 36 and 52 min, respectively. Endogenous proteins and peroxides were blocked using Ventana pre-peroxidase inhibitor solution. The slides were then incubated with the following primary antibodies: rabbit monoclonal anti-HIF-1 α [clone: EP1215Y] (Abcam, Cambridge, UK, Cat# ab51608, dilution 1:100) for 44 min at 37 °C; rabbit polyclonal anti-β3-AR (Abcam, Cambridge, UK, Cat# ab94506, dilution 1:100) for 44 min at 37 °C; and mouse monoclonal anti-CD31 [clone: JC70] (Ventana Medical Systems, Inc., Basel, Switzerland, Cat# 05463475001, ready to use) for 32 min at 37 °C. The sections were then counterstained with hematoxylin (Ventana Medical Systems, Inc., Basel, Switzerland, Cat# 07024282001) for 12 min, followed by a post-counterstain bluing step (Ventana Medical Systems, Inc., Basel, Switzerland, Cat# 07024282001) for 4 min, and then mounted. Negative controls were performed by replacing the primary antibody with non-immune rabbit or mouse serum. Immunostaining was independently assessed by three pathologists (C.S., G.N.F., and A.G.N.), who were blinded to clinicopathological data. Discrepancies were resolved by joint review and consensus.

The percentage of positive cells was assessed within the neoplastic area, irrespective of staining intensity, which was not included in the scoring system because of its limited reproducibility across cases and cellular compartments; instead, the proportion of positive cells provided a more consistent and interpretable parameter for comparative analysis. HIF-1α was scored as cytoplasmic and nuclear staining and β3-AR was scored as cytoplasmic staining. Biomarker evaluation was performed in a compartment-specific manner: β3-AR expression was assessed separately in macrophages and melanocytes, whereas HIF-1α expression was evaluated separately in melanocytes and TILs. Compartment-specific positivity was assigned according to the morphological identification of the stained cell population on the corresponding immunostained section. MART-1 immunostaining was additionally used to support the identification and distribution of melanocytic cells.

Angiogenesis was assessed by measuring MVD using CD31, an endothelial cell marker, and its expression was evaluated digitally. Histological preparations were digitally acquired with a Leica Aperio AT2 scanner (Leica, Wetzlar, Germany). For each case, the regions of interest (ROIs) comprising the entire lesion were annotated using the HALO image analysis software (version 3.5.3577, IndicaLabs Albuquerque, NM, USA). The area of CD31-positive cells was then evaluated as the percentage of the neoplastic region using the Area Quantification Brightfield Algorithm (v 2.2) for each ROI rather than as a conventional vessel count-based MVD measure. To optimize the evaluation, staining artifacts and non-specific signals were manually excluded. Although the use of AEC/red chromogen reduced direct overlap with melanin pigmentation compared with brown chromogens, algorithm thresholds were manually adjusted for each slide to minimize pigment-related or background-related false-positive detection.

### 2.3. Statistical Analysis

For statistical analysis, among the clinical and histopathological characteristics observed, the selected variables (*vide infra*) were present in at least 10% of the study population (minimum cutoff: 3 ID). The pathological variables analyzed included melanocytic lesion type, ulceration, and TILs, whereas the dermoscopic variables were blue-white veil, regression, atypical network, and ulceration. The continuous variables are summarized as the mean ± standard deviation (SD). Before applying parametric tests, the distribution of continuous variables was assessed and found to be approximately Gaussian, with the median always falling within the 95% confidence interval. Therefore, parametric methods were considered appropriate for the planned exploratory analyses. To compare the number (expressed as percentage) of positive cells per lesion after immunological staining—including morphologically identified macrophages—among the groups (dysplastic nevi, in situ primary melanoma, pT1a melanomas, and >pT1a melanomas), a one-way ANOVA followed by multiple comparisons with Fisher’s least significant difference (LSD) method was performed. Furthermore, the comparison of the number of positive cells with other categorical clinical, pathological (TILs and histopathological ulceration), and dermoscopic variables (blue-white veil, ulceration, atypical network, and regression) was assessed using *t*-test for independent samples (two-tailed). Relationships between biomarkers were explored using Pearson correlation analysis. Because this study was designed as an exploratory, retrospective analysis of a limited number of well-characterized archival specimens, the statistical analyses were primarily performed to identify potential biological patterns rather than to provide confirmatory evidence. Consequently, Fisher’s LSD test was selected to preserve sensitivity for detecting potentially relevant differences across biologically defined groups. No formal correction for multiple comparisons was applied, as more conservative approaches would have substantially reduced statistical power and increased the risk of type II errors in this limited dataset. Accordingly, all statistical findings were interpreted with appropriate caution and should be considered hypothesis-generating. Statistical significance was set at 0.05, and all analyses were conducted using IBM SPSS Statistics (v. 28) (IBM Corp., Armonk, NY, USA).

## 3. Results

### 3.1. Study Population

The patients’ clinicopathological features are shown in Table 1. Within the group of 27 patients recruited in the present study (14 males and 13 females), the mean age was 61.1 ± 15.5 years (yrs). Dysplastic nevi and in situ primary melanomas were more common among patients with an average age of 52.4 ± 15.3 yrs. In contrast, an incidence shift in older age (sixth and seventh decade) was observed in invasive melanoma lesions (mean age: 69.1 ± 11.1 yrs). No case of melanoma was observed in patients younger than 50 yrs. Histopathological melanoma subtypes included superficial spreading melanoma in situ (*n* = 6), superficial spreading invasive melanoma (*n* = 10), nodular melanoma (*n* = 2) and lentigo maligna melanoma (*n* = 2). Dermoscopic features, such as ulceration, blue-white veil, regression, and atypical network, are reported in Table 1 for each patient, grouped by melanocytic lesion type and anatomical localization.

### 3.2. Immunohistochemical Marker Expression and Its Association with Clinicopathological Findings

#### 3.2.1. HIF-1 α, β3-AR and CD31 Expression in Tissue Samples

**Figure 1:**
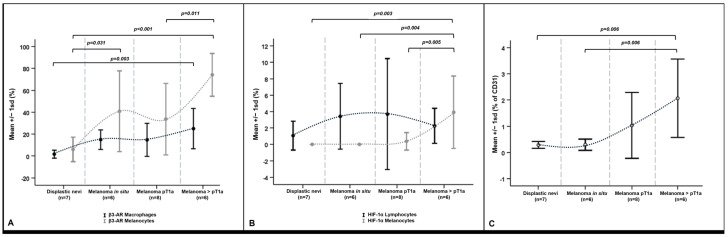
**Dot and whisker plot of marker expression in cutaneous melanocytic lesions for each cellular subset in different lesion groups**. β3-AR expression in macrophages and melanocytes (**A**), HIF-1α expression in lymphocytes and melanocytes (**B**), and CD31 expression (**C**). Data are summarized as mean ± SD of *n* = 7 dysplastic nevi; *n* = 6 melanoma in situ; *n* = 8 melanoma pT1a, and *n* = 6 melanoma >pT1a. Statistically significant groups comparisons and trend lines are plotted.

**Figure 2:**
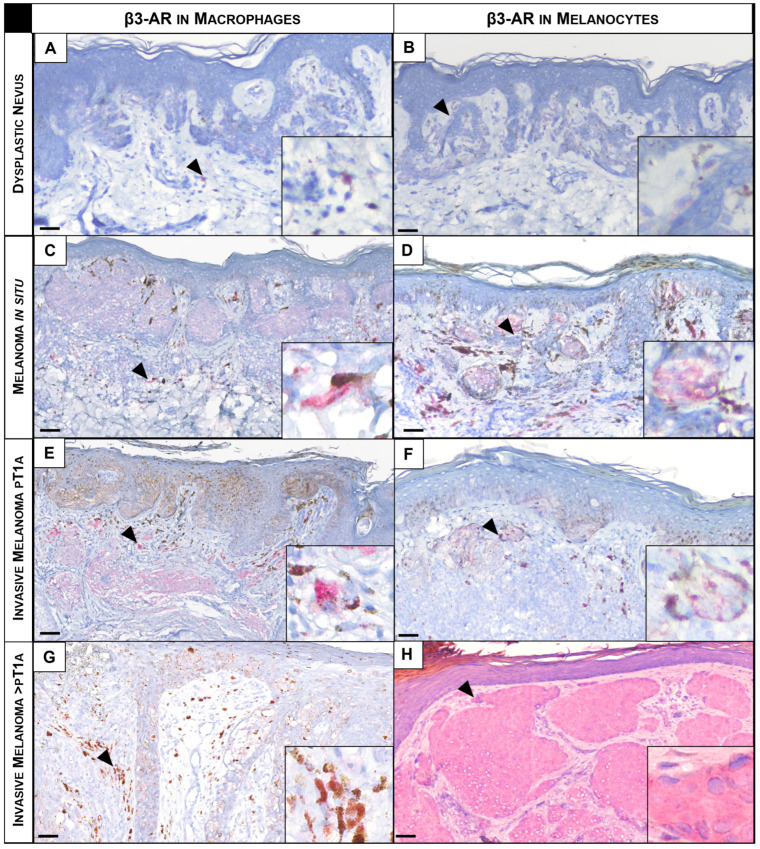
**Representative images of β3-AR expression (in red) in macrophages and melanocytes in different lesion groups**. Dysplastic nevi (**A**,**B**), melanoma in situ (**C**,**D**), melanoma pT1a (**E**,**F**) and melanoma >pT1a (**G**,**H**). Higher magnification insets that highlight macrophage-enriched areas for each lesion are presented in (**A**,**C**,**E**,**G**). Higher magnification insets that highlight β3-AR cytoplasmic expression in melanocytes for each lesion are presented in (**B**,**D**,**F**,**H**). Arrowheads point at representative macrophages and melanocytes expressing β3-AR (in red). Magnification for all images: 200×. Magnification for all insets images: 400×. Scale bar: 50 μm.

The β3-AR expression, evaluated in conjunction with HIF-1α and CD31 expression, and its possible pathological correlations with different types of melanocytic lesions were investigated. Figure 1 and Table 2 provide a summary of the percentages of different cell types with positive staining and a comparison among lesion groups. β3-AR was expressed at different levels across all lesion groups and was always confined to the cytoplasm of macrophages and melanocytes (Figure 2 and Figures S1 and S2). Significantly higher expression of β3-AR was observed in macrophages of the invasive >pT1a melanoma group (Figure 1A and Figure 2G) and melanocytes of the in situ (Figure 1A and Figure 2D) and >pT1a melanoma groups (Figure 1A and Figure 2H) compared to the dysplastic nevus group (Figure 1A and Figure 2A,B) and in melanocytes of the melanoma pT1a group (Figure 1A and Figure 2F) versus >pT1a melanoma group (Figure 1A and Figure 2H), respectively.

**Figure 3:**
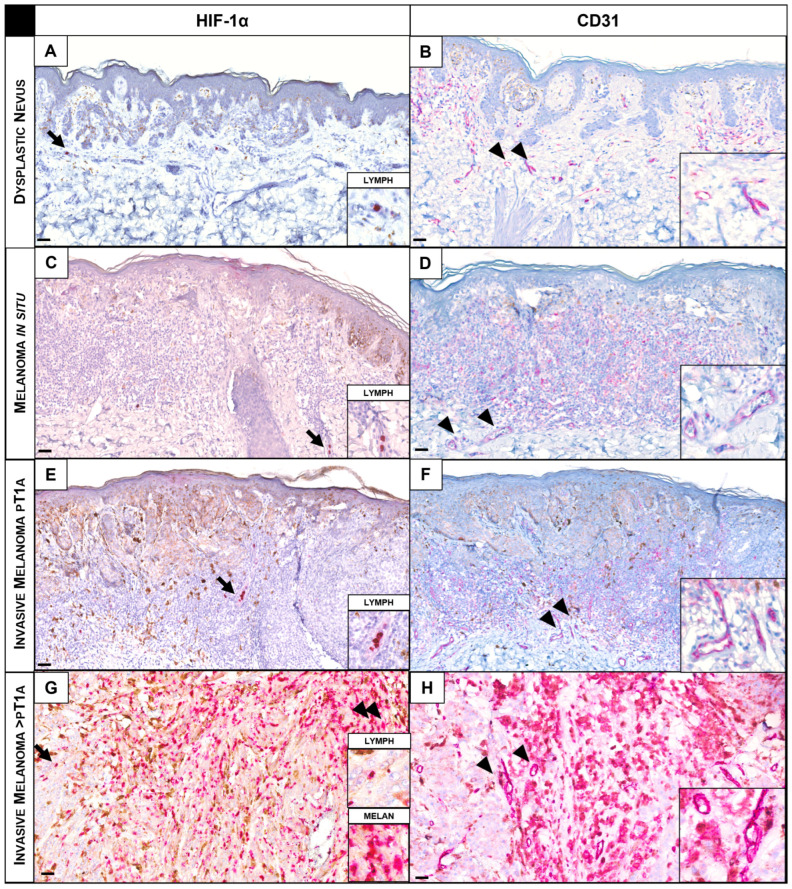
**Representative images of HIF-1α and CD31 expression (in red) in lymphocytes, melanocytes and endothelial cells in different lesion groups**. Dysplastic nevus (**A**,**B**), melanoma in situ (**C**,**D**), melanoma pT1a (**E**,**F**) and melanoma >pT1a (**G**,**H**). Higher magnification insets that highlight HIF-1α cytoplasmic expression in lymphocytes are presented in (**A**,**C**,**E**) and expression in melanocytes is presented in (**G**). Arrows point at representative lymphocytes expressing HIF-1α. Arrowheads point at representative melanocytes expressing HIF-1α (in red). Higher magnification insets that highlight vascular enriched areas and CD31 expression in endothelial cells for each lesion are presented in (**B**,**D**,**F**,**H**). Arrowheads point at representative vessels expressing CD31 (in red). Magnification for all images: 200×. Magnification for all insets images: 400×. Scale bar: 50 μm.

Although HIF-1α expression, which was consistently restricted to the cytoplasm of lymphocytes and melanocytes with no detectable nuclear staining (Figure 3 and Figure S3), remained largely unchanged in TILs, markedly lower expression of HIF-1α in the melanocytes of dysplastic nevi (Figure 1B and Figure 3A), and in situ (Figure 1B and Figure 3C) and pT1a melanomas (Figure 1B and Figure 3E) was observed compared to >pT1a melanomas (Figure 1B and Figure 3G) (Table 2).

Finally, representative CD31 IHC pictures of melanocytic lesions are shown in Figure 3. A higher MVD was present in the invasive >pT1a melanoma group (Figure 1C and Figure 3H) compared to the dysplastic nevus (Figure 1C and Figure 3B) and in situ groups (Figure 1C and Figure 3D), whereas there were no significant differences in CD31 expression versus the pT1a melanoma group (Figure 1C and Figure 3F) (Table 2).

#### 3.2.2. Comparison Between Expression Markers, Clinicopathological, and Dermoscopic Features

Comparisons of marker expression and different clinicopathological features of the pT1a and >pT1a melanoma groups in the studied cohort were performed.

No statistically significant associations were found between β3-AR, HIF-1α, and CD31 expression and TILs and histopathological ulceration across the different cellular subsets. Although an increasing trend was observed, our preliminary data suggested that the presence or absence of TILs did not alter the β3-AR expression in macrophages and melanocytes in the lesions with infiltrating lymphocytes (*n* = 11) compared to non-infiltrated melanocytic lesions (*n* = 3), possibly due to the small sample size for the latter category (Table S1). Similar results were observed for HIF-1α and CD31 expression.

Likewise, there were no significant differences in β3-AR expression between macrophages and melanocytes in the melanocytic lesions with histopathological ulceration. On the other hand, HIF-1α was markedly and significantly overexpressed in the melanocytes of ulcerated lesions compared to non-ulcerated lesions (*p* = 0.007); CD31 levels were also higher, but the difference did not reach statistical significance (Table S1). Dermoscopic variables in both the pT1a and >pT1a melanoma groups were explored as descriptive clinical parameters, and their potential association with biomarker expression was investigated in an exploratory, hypothesis-generating manner. However, no statistically significant correlations were observed between dermoscopic features and the expression of β3-AR, HIF-1α, or CD31. Therefore, these findings should be considered descriptive and interpreted with caution given the limited sample size of the study. The present results do not allow for firm conclusions regarding the relationship between dermoscopic features and the expression of β3-AR, HIF-1α, or CD31. Further studies in larger cohorts are warranted to clarify the biological relevance of these observations.

#### 3.2.3. Biomarker Correlation Analysis

A correlation analysis was performed on the different cellular subsets (macrophages and melanocytes) within the entire patient cohort to further characterize the relationships among biomarkers. The results showed a direct correlation, as indicated by a positive Pearson correlation coefficient (r), which reflects its strength.

**Figure 4:**
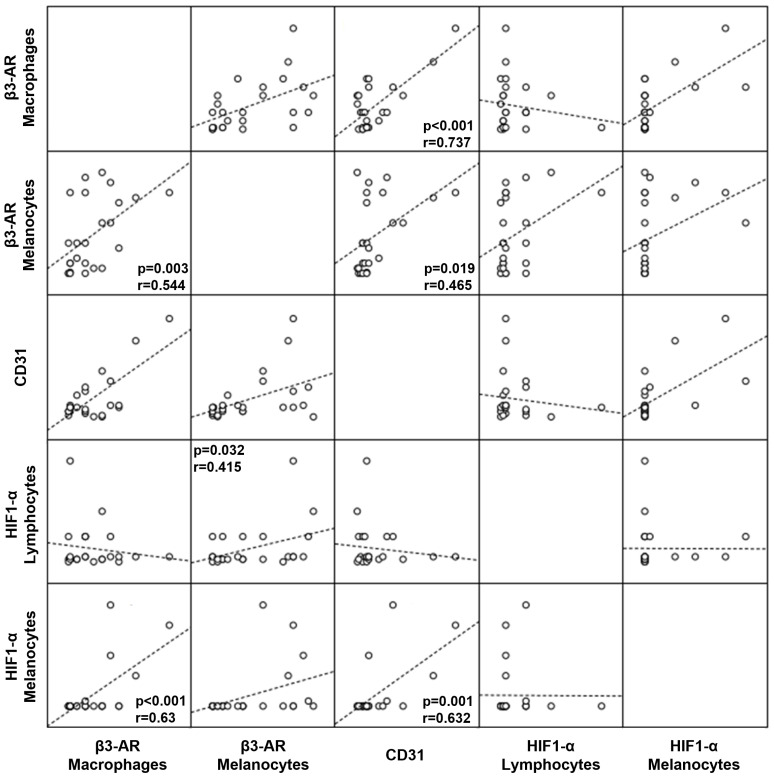
**Scatter plot matrix visualizing the relationships between three markers (β3-AR, HIF-1 α, and CD31) within three cellular subsets (macrophages, melanocytes, and lymphocytes) in the whole patient cohort.** Correlations are represented starting from the *x*-axis and going up. Open circles represent individual patient samples, and dashed lines indicate the linear regression trend. Significant correlations, indicated with *p*-values (0.05 level, 2-tailed) and correlation coefficients (r) are plotted.

β3-AR expression in macrophages was positively related to HIF-1α and β3-AR expression in melanocytes (*p* < 0.001, r = 0.63; *p* = 0.003, r = 0.544, respectively). In addition, the levels of β3-AR expression in melanocytes were positively related to HIF-1α expression in lymphocytes (*p* = 0.032; r = 0.415) (Figure 4, Table S2).

Furthermore, MVD was positively and significantly correlated with β3-AR expression in macrophages (*p* < 0.001, r = 0.737) and in melanocytes (*p* = 0.019, r = 0.465), and with hypoxia marker HIF-1α expression in melanocytes (*p* = 0.001, r = 0.632) (Figure 4, Table S2).

## 4. Discussion

New therapies for metastatic melanoma have significantly improved patient survival, mainly through immunotherapy and targeted approaches [9]. Experimental evidence indicates that β3-ARs regulate VEGF release via NO production and promote pro-inflammatory cytokine secretion, thereby supporting hypoxia-driven angiogenesis and melanoma aggressiveness, suggesting a potential antimetastatic role [14]. However, the lack of selective β3-AR antagonists and interspecies differences in receptor expression limit the current translational relevance [24]. Consequently, the clinical role of β3-AR in melanoma remains unclear, warranting the development of more selective pharmacological tools. Notably, β3-AR activity in stromal cells—which are less prone to resistance—highlights β3-AR blockade as a potential strategy to overcome resistance mechanisms.

Our study showed that β3-AR was expressed in human melanocytic lesions, with a strong up-regulation in invasive malignant lesions, supporting its involvement in aggressive disease. However, the observed pattern of β3-AR expression being significantly higher in melanoma in situ and >pT1a, but not in pT1a melanoma, might reflect dynamic changes in the tumor microenvironment during progression.

As tumors progressed from in situ to invasive stages, they experienced varying levels of hypoxia within the tumor microenvironment. β3-AR expression is also upregulated under hypoxic conditions as part of the tumor’s adaptive mechanism [25]. In melanoma in situ, early hypoxic conditions may contribute to increased β3-AR expression, whereas during transitions to pT1a lesions, more heterogeneous or less pronounced hypoxia may be associated with relatively lower expression levels. In more advanced stages (>pT1a), a progressively hypoxic tumor microenvironment may again favor increased β3-AR expression. Importantly, early β3-AR expression may also be influenced by hypoxia-independent mechanisms, such as enhanced adrenergic signaling in neoplastic tissues [11,13]. In this context, the higher β3-AR expression observed in melanoma in situ and in more advanced lesions (>pT1a) in our cohort may reflect changes in the tumor microenvironment associated with hypoxia-related signaling. However, the present study was not designed to directly investigate the regulatory mechanisms underlying β3-AR expression. Therefore, the observed expression pattern should be interpreted with caution and may reflect an association rather than a causal relationship.

These findings are consistent with increased HIF-1α expression in malignant melanocytes and elevated CD31 expression in >pT1a melanomas, supporting a potential relationship among hypoxia, the adrenergic response, and tumor vascularization. The higher HIF-1α expression observed in advanced melanomas (>pT1a) might suggest an increased stabilization/accumulation of the protein in a more hypoxic and metabolically stressed microenvironment during tumor progression, leading to a robust activation of pro-angiogenic pathways, including the VEGF and β3-AR pathways [20]. Indeed, previous studies have reported a positive correlation between HIF-1α expression and the degree of malignancy, particularly in cutaneous melanoma invading the dermis [19]. Accordingly, our findings may suggest that β3-AR expression is associated with microenvironmental changes occurring during melanoma progression. In line with these observations, accumulating evidence indicates that hypoxia-driven signaling also contributes to melanoma cellular plasticity, stemness, and therapy resistance [26]. In particular, HIF-1α has been implicated in phenotypic switching toward a more invasive, stem-like state, which is being increasingly recognized as a mediator of therapeutic resistance in cutaneous melanoma [27]. Moreover, tumor microenvironmental cues—including stress-related adrenergic signaling—may promote tumor adaptation and resistance to both targeted therapies and immune checkpoint inhibitors. Recent studies have highlighted the central role of the melanoma microenvironment in orchestrating these adaptive responses through dynamic interactions among tumor cells, stromal components, immune cells, and hypoxia-related signaling pathways [28]. Although these mechanisms were not directly investigated in the present study, the observed association between β3-AR, HIF-1α, and CD31 is consistent with the hypothesis that these pathways may participate in the complex biological networks underlying melanoma progression. Further functional studies will be required to clarify these potential relationships and their possible translational relevance.

Furthermore, our study confirmed the strong correlation between CD31 and stress- and hypoxia-related markers, consistent with previous reports linking increased CD31 expression to vasculogenic mimicry and neoangiogenesis in aggressive melanoma [22]. However, the lack of statistically significant differences between pT1a and >pT1a melanomas, as well as between pT1a and in situ lesions, despite the observed trends and the higher CD31 expression in advanced melanomas, may reflect biological overlap in angiogenic profiles across intermediate stages. Indeed, angiogenesis does not progress uniformly throughout melanoma development; despite dramatic vascular remodeling during tumor growth, the overall proangiogenic gene expression profile may remain relatively constant during the early (in situ/pT1a) and intermediate stages (e.g., pT2–3). Thus, as in our study, when comparing the extreme ends of the spectrum, subtle differences present within intermediate stages can be overlooked. In this context, ulcerated lesions showed a trend toward increased β3-AR and CD31 expression in macrophages and melanocytes, together with significantly higher HIF-1α expression in melanocytes. Since histopathological ulceration is a well-established adverse prognostic factor that is incorporated into the AJCC 8th Edition staging system [23], our findings are consistent with the hypothesis that hypoxia-driven angiogenic remodeling becomes more prominent in biologically aggressive melanomas.

Although TILs reflect host immune response in patients with melanoma, their prognostic value in melanoma and their incorporation into the AJCC staging system remain under debate. In our cohort, TILs’ presence did not significantly influence β3-AR, HIF-1α, or CD31 expression, possibly due to the sample size limitations and biological heterogeneity. Likewise, no significant associations were observed between dermoscopy and biomarker expression. Given the limited cohort size, the present study was not designed to establish correlations between dermoscopic patterns and the molecular alterations investigated. Therefore, no definitive conclusions can be drawn regarding the relationship between dermoscopic patterns and the expression of β3-AR, HIF-1α, or CD31. Nevertheless, dermoscopy remains a valuable non-invasive tool for characterizing melanoma morphology, and future multimodal studies combining dermoscopy, digital pathology, and molecular profiling may help clarify whether distinct dermoscopic phenotypes correspond to specific microenvironmental states during melanoma progression.

Taken together, the strong correlation among β3-AR, HIF-1α, and CD31 expression supports a potential interplay between stress signaling, hypoxia, and neovascularization in melanoma, in agreement with previous experimental evidence [18], suggesting that β3-AR may be associated with hypoxia-related changes in the tumor microenvironment.

β3-AR signaling in melanoma has been linked to multiple downstream pathways, including Gs/cAMP/PKA-mediated cytokine and VEGF release [17], and PI3K/AKT, STAT3, iNOS 15 [15], and endothelial NOS (eNOS/NOS-3) activation [18]. However, determining the specific intracellular mechanism remains challenging, and the present study was not designed to investigate these pathways directly and therefore provides observational rather than mechanistic evidence. Additionally, lymphocytes are not typically the primary source of HIF-1α signals in melanoma. Instead, HIF-1α primarily acts within tumor and stromal cells, modulating the microenvironment through angiogenic and immunomodulatory factors. Hypoxia also alters immune cell composition and function, potentially by converting immune infiltrates into tumor-supportive elements [29]. Consistently, inhibition of HIF-1α activity has been shown to restore immune permissiveness by enhancing NK and CD8+ T-cell infiltration in melanoma [30].

Importantly, β3-AR overexpression is not restricted to cancer cells; in macrophages under hypoxic conditions, it is likely a response to the altered tumor microenvironment, where it may promote monocyte recruitment, macrophage polarization, and pro-tumorigenic function. Calvani et al. reported that only M2-polarized macrophages, not M1-polarized ones, contact and induce β3-AR expression in cancer cells [14]. It is also plausible that β3-ARs may modulate melanoma-associated inflammation. Indeed, catecholamine stimulation could enhance inflammatory cytokine release, sustaining β2/β3-AR activation and tumor progression [14]. Collectively, these observations suggest that β3-ARs may serve as mediators of tumor–microenvironment interactions in melanoma. However, the present study does not provide functional evidence for these mechanisms, and further investigations are required to clarify their role in melanoma progression.

As with most studies, the design of the current investigation is subject to limitations that should be considered when interpreting the findings. Immune cell subsets were assigned based on morphological features and were further supported by MART-1 immunostaining to identify the melanocytic component. However, the absence of cell-specific markers (e.g., CD68, CD3, or CD45) precluded unequivocal identification of immune cell populations. In addition, the retrospective design, the relatively sample size constraints and the subdivision of patients into multiple pathological groups reduced the statistical power for certain subgroup analyses. Furthermore, the >pT1a category included biologically heterogeneous lesions (pT2–pT4), which may have masked potential stage-specific differences. Because the study was based on retrospectively available samples, no formal power analysis was performed. Moreover, given the exploratory nature of the study and the limited sample size, the statistical analyses were primarily intended to identify potential patterns of biomarkers rather than to provide confirmatory evidence. Accordingly, the results should be interpreted cautiously, considered hypothesis-generating, and validated in larger cohorts. An additional limitation concerns the digital assessment of CD31. Although the use of AEC/red chromogen reduced direct overlap with melanin pigmentation, residual pigment- or background-related interference cannot be completely excluded. Therefore, despite manual artifact exclusion and slide-by-slide threshold adjustment, the CD31-positive area should be interpreted cautiously as a semi-quantitative surrogate for vascularization. Finally, correlation of β3-AR or HIF-1α expression with patient metastasis, relapse, other clinical parameters, or treatment regimens or responses was not evaluated. The current preliminary study focused on characterizing the expression of the aforementioned markers in tissue samples and on correlational analyses rather than on functional assays investigating the underlying molecular mechanism. Therefore, future functional studies are needed to validate these preliminary observations, provide deeper insights into the mechanistic role of β3-AR and its potential regulation by HIF-1α in melanoma cells and define their potential biological and translational relevance.

In conclusion, while preclinical studies have highlighted the potential benefits of β1/2-AR blockers in melanoma [31], our preliminary findings suggest that β3-adrenoceptors may represent a pathway of biological interest in the melanoma microenvironment. Advances in nanotechnology, particularly ultrabright theranostic delivery systems, may enable selective targeting of β3-AR-mediated pathways, thereby enhancing efficacy while limiting systemic toxicity [32]; this approach has already been explored in the treatment of vascular diseases [33]. However, although these observations provide a biological rationale for further investigation of β3-AR-related pathways, the therapeutic implications remain speculative and require validation in dedicated functional and clinical studies.

These findings highlight a potential association between β3-AR signaling, hypoxia-related pathways, and tumor vascularization in melanoma, supporting the need for further mechanistic studies to clarify their role in melanoma progression.

## Figures and Tables

**Table 1:** Clinical-pathological and dermoscopic features of patients.

Patient	Gender	Age (Years)	Diagnosis	Histopathological Subtype	Location	Breslow Thickness (mm)	Tumor-Infiltrating Lymphocytes	Histopathological Ulceration	T Stage (AJCC 8th Ed.)	Dermoscopic Features
1	M	39	Dysplastic nevus	NA	Back	NA	NA	NA	NA	Regression Irregular globes Atypical network
2	F	28	Dysplastic nevus	NA	Lower leg	NA	NA	NA	NA	Atypical network
3	F	55	Dysplastic nevus	NA	Back	NA	NA	NA	NA	Atypical network
4	M	68	Dysplastic nevus	NA	Lower leg	NA	NA	NA	NA	Atypical network
5	F	77	Dysplastic nevus	NA	Back	NA	NA	NA	NA	Regression Irregular globes Atypical network
6	F	40	Dysplastic nevus	NA	Back	NA	NA	NA	NA	Regression Irregular globes Atypical network
7	F	63	Dysplastic nevus	NA	Abdomen	NA	NA	NA	NA	Atypical network
8	M	52	Melanoma in situ	Superficial spreading melanoma in situ	Lower leg	0	NA	NA	Tis	Regression Atypical network
9	M	68	Melanoma in situ	Superficial spreading melanoma in situ	Back	0	NA	NA	Tis	Atypical network Blue-white veil
10	M	51	Melanoma in situ	Superficial spreading melanoma in situ	Trunk	0	NA	NA	Tis	Irregular globes
11	M	57	Melanoma in situ	Superficial spreading melanoma in situ	Abdomen	0	NA	NA	Tis	Regression Atypical network Blue-white veil
12	F	27	Melanoma in situ	Superficial spreading melanoma in situ	Lower leg	0	NA	NA	Tis	Regression Atypical network
13	F	57	Melanoma in situ	Superficial spreading melanoma in situ	Upper limb	0	NA	NA	Tis	Atypical network
14	M	81	Invasive melanoma	Superficial spreading invasive melanoma	Trunk	0.7	Present	Absent	pT1a	Regression Reverse network
15	M	60	Invasive melanoma	Lentigo maligna melanoma	Head	0.4	Present	Absent	pT1a	Atypical network
16	F	80	Invasive melanoma	Lentigo maligna melanoma	Upper limb	0.4	Absent	Absent	pT1a	Atypical network
17	M	63	Invasive melanoma	Superficial spreading invasive melanoma	Upper limb	0.4	Present	Absent	pT1a	Regression Atypical network Blue-white veil
18	M	64	Invasive melanoma	Superficial spreading invasive melanoma	Lower leg	0.7	Present	Absent	pT1a	Atypical network
19	F	71	Invasive melanoma	Superficial spreading invasive melanoma	Upper limb	0.2	Present	Absent	pT1a	Atypical network
20	M	68	Invasive melanoma	Superficial spreading invasive melanoma	Upper limb	0.5	Present	Absent	pT1a	Atypical network Blue-white veil
21	M	83	Invasive melanoma	Superficial spreading invasive melanoma	Upper limb	0.5	Present	Absent	pT1a	Regression Atypical network
22	M	56	Invasive melanoma	Superficial spreading invasive melanoma	Upper limb	1.3	Absent	Absent	pT2a	Regression Atypical network Blue-white veil Atypical vessels
23	M	86	Invasive melanoma	Superficial spreading invasive melanoma	Lower leg	3	Present	Present	pT3b	Regression Atypical network Blue-white veil Atypical vessels Ulceration
24	F	56	Invasive melanoma	Nodular melanoma	Lower leg	2.4	Present	Absent	pT3a	Atypical network Blue-white veil Ulceration
25	F	52	Invasive melanoma	Superficial spreading invasive melanoma	Upper limb	1.1	Present	Absent	pT2a	Irregular globes Atypical network
26	F	70	Invasive melanoma	Superficial spreading invasive melanoma	Lower leg	3.3	Present	Present	pT3a	Regression Atypical network Blue-white veil
27	F	77	Invasive melanoma	Nodular melanoma	Back	17	Absent	Present	pT4a	Ulceration

NA: not applicable; M: male; F: female.

**Table 2:** Immunohistochemical marker expression in cutaneous human melanocytic lesions: percentage of positive cells in each lesion.

Biomarker	Cellular Subset	Lesion Group	Mean ± SD	*p*-Value (ANOVA)	Group Comparison	*p*-Value (*t*-Test)
**β3-AR**	**Macrophages**			*0.028* *	Dysplastic nevi vs. Melanoma in situ	0.075
		Dysplastic nevi	1.71 ± 3.68		Dysplastic nevi vs. Melanoma pT1a	0.061
		Melanoma in situ	15.00 ± 8.94		Dysplastic nevi vs. Melanoma >pT1a	*0.003* *
		Melanoma pT1a	14.75 ± 15.06		Melanoma in situ vs. Melanoma pT1a	0.971
		Melanoma >pT1a	25.00 ± 18.44		Melanoma in situ vs. Melanoma >pT1a	0.189
					Melanoma pT1a vs. Melanoma >pT1a	0.152
	**Melanocytes**			*0.002* *	Dysplastic nevi vs. Melanoma in situ	0.031 *
		Dysplastic nevi	6.00 ± 11.18		Dysplastic nevi vs. Melanoma pT1a	0.061
		Melanoma in situ	40.83 ± 36.93		Dysplastic nevi vs. Melanoma >pT1a	<0.001 *
		Melanoma pT1a	33.75 ± 32.71		Melanoma in situ vs. Melanoma pT1a	0.634
		Melanoma >pT1a	74.17 ± 19.60		Melanoma in situ vs. Melanoma >pT1a	0.045
					Melanoma pT1a vs. Melanoma >pT1a	0.011 *
**HIF-1α**	**Lymphocytes**			0.666	Dysplastic nevi vs. Melanoma in situ	0.346
		Dysplastic nevi	1.07 ± 1.77		Dysplastic nevi vs. Melanoma pT1a	0.261
		Melanoma in situ	3.42 ± 4.01		Dysplastic nevi vs. Melanoma >pT1a	0.633
		Melanoma pT1a	3.69 ± 6.76		Melanoma in situ vs. Melanoma pT1a	0.910
		Melanoma >pT1a	2.25 ± 2.14		Melanoma in situ vs. Melanoma >pT1a	0.649
					Melanoma pT1a vs. Melanoma >pT1a	0.550
	**Melanocytes**			*0.009* *	Dysplastic nevi vs. Melanoma in situ	1.000
		Dysplastic nevi	0.00 ± 0.00		Dysplastic nevi vs. Melanoma pT1a	0.748
		Melanoma in situ	0.00 ± 0.00		Dysplastic nevi vs. Melanoma >pT1a	0.003 *
		Melanoma pT1a	0.38 ± 1.06		Melanoma in situ vs. Melanoma pT1a	0.748
		Melanoma pT1a	0.38 ± 1.06		Melanoma in situ vs. Melanoma >pT1a	0.004 *
					Melanoma pT1a vs. Melanoma >pT1a	0.005 *
**CD31**				*0.017* *	Dysplastic nevi vs. Melanoma in situ	0.984
		Dysplastic nevi	0.28 ± 0.13		Dysplastic nevi vs. Melanoma pT1a	0.195
		Melanoma in situ	0.30 ± 0.21		Dysplastic nevi vs. Melanoma >pT1a	0.006 *
		Melanoma pT1a	1.03 ± 1.26		Melanoma in situ vs. Melanoma pT1a	0.202
		Melanoma >pT1a	2.07 ± 1.50		Melanoma in situ vs. Melanoma >pT1a	0.006 *
					Melanoma pT1a vs. Melanoma >pT1a	0.076

Patients were categorized into four groups: dysplastic nevi (*n* = 7), melanoma in situ (*n* = 6), melanoma pT1a (*n* = 8) and melanoma >pT1a (*n* = 6). Values in bold italics indicate statistically significant results. Group differences were considered statistically significant at * *p*-value < 0.05. The continuous variables are summarized as the mean ± standard deviation (SD).

## Data Availability

The data that support the findings of this study are available from the corresponding author upon reasonable request.

## Supplementary Materials

The following supporting information can be downloaded at: https://www.mdpi.com/article/10.3390/dermatopathology13030031/s1, Table S1: Comparison between expression markers and different clinical-pathological features in pT1a and >pT1a melanoma groups; Table S2: Matrix of Pearson Correlation analysis; Figure S1: Representative images of H&E and MART-1 staining (in red) from the cases shown in Figure 2, (A, C, E, G) corresponding to the analysis of β3-AR expression in macrophages. Dysplastic nevus, H&E (A) and corresponding MART-1 staining (B), Melanoma in situ, H&E (C) and corresponding MART-1 staining (D), Melanoma pT1a, H&E (E) and corresponding MART-1 staining (F), and Melanoma >pT1a, H&E (G) and corresponding MART-1 staining (H). Please note that, for some samples, MART-1 staining was performed on serial sections from the same FFPE block or on sections from additional FFPE blocks of the same lesion; therefore, the images may not strictly correspond to the exact sections shown in Figure 2, Figure S2: Representative images of H&E and MART-1 staining (in red) from the cases shown in Figure 2 (B, D, F, H) corresponding to the analysis of β3-AR expression in melanocytes. Dysplastic nevus, H&E (A) and corresponding MART-1 staining (B), Melanoma in situ, H&E (C) and corresponding MART-1 staining (D), Melanoma pT1a, H&E (E) and corresponding MART-1 staining (F), and Melanoma >pT1a, H&E (G) and corresponding MART-1 staining (H). Please note that, for some samples, MART-1 staining was performed on serial sections from the same FFPE block or on sections from additional FFPE blocks of the same lesion; therefore, the images may not strictly correspond to the exact sections shown in Figure 2, Figure S3: Representative images of H&E and MART-1 staining (in red) from the cases shown in Figure 3. Dysplastic nevi (A, B), Melanoma in situ (C, D), Melanoma pT1a (E, F), and Melanoma >pT1a (G, H). Please note that, for some samples, MART-1 staining was performed on serial sections from the same FFPE block or on sections from additional FFPE blocks of the same lesion; therefore, the images may not strictly correspond to the exact sections shown in Figure 3.

## Abbreviations

The following abbreviations are used in this manuscript:

HIF-1α	Hypoxia-inducible factor 1-alpha
β3-AR	β3-adrenergic receptor
TILs	Tumor-infiltrating lymphocytes
MVD	Microvascular density
FFPE	Formalin-fixed, paraffin-embedded
IHC	Immunohistochemistry
