# Supplementary material for: Exploring β3-Adrenergic Receptor, HIF-1α, and CD31 Interplay in the Microenvironment of Atypical Melanocytic Lesions
Source: Dermatopathology (Basel). 2026 Jul 3;13(3):31. doi: 10.3390/dermatopathology13030031 (PMC13398111; doi:10.3390/dermatopathology13030031)
Supplement: Supplementary file 1 [file dermatopathology-13-00031-s001.zip › dermatopathology-4400497-supplementary.pdf]

Supplementary materials

Exploring β3-Adrenergic receptor, HIF-1α, and CD31 interplay in the microenvironment of atypical melanocytic lesions

Eugenia Belcastro [1, *, †], Giuseppe Nicolò Fanelli [2,3,4,†], Cristian Fidanzi [5,6,7], Desirèe Fischetti [5], Riccardo Morganti [8], Katia De Ieso [3], Luca Filippi [7,9], Antonio Giuseppe Naccarato [2,3], Marco Romanelli [5,7], Cristian Scatena [2,3,#], Agata Janowska [5,7,#]

[1] Department of Translational Research and New Technologies in Medicine and Surgery, University of Pisa, 56126, Pisa, Italy. eugenia.belcastro@unipi.it

[2] Division of Pathology, Department of Translational Research and New Technologies in Medicine and Surgery, University of Pisa, 56126, Pisa, Italy. nicolo.fanelli@unipi.it or gif4002@med.cornell.edu; giuseppe.naccarato@unipi.it; cristian.scatena@unipi.it

[3] Department of Oncology, Pisa University Hospital, 56126, Pisa, Italy. nicolo.fanelli@unipi.it; ka.deieso@ao-pisa.toscana.it

[4] Department of Pathology and Laboratory Medicine, Weill Cornell Medicine, New York, NY, 10021, USA.

[5] Unit of Dermatology, Pisa University Hospital, 56126, Pisa, Italy. cri.fidanzi@outlook.it; d.fischetti@studenti.unipi.it; marco.romanelli@unipi.it; agata.janowska@unipi.it

[6] Melanoma and Skin Cancer Unit AVNO (Area Vasta Nord Ovest) Tuscany, Carrara Hospital, 54033, Carrara, Italy.

[7] Department of Clinical and Experimental Medicine, University of Pisa, 56126, Pisa, Italy. luca.filippi@meyer.it

[8] Statistics Unit, Pisa University Hospital, 56126, Pisa, Italy. r.morganti@ao-pisa.toscana.it

[9] Neonatal Intensive Care Unit, Meyer Children's Hospital IRCCS, 50139, Florence, Italy.

† Eugenia Belcastro and Giuseppe Nicolò Fanelli should be considered joint first author.

# Cristian Scatena and Agata Janowska should be considered joint senior author.

* Correspondence:
Eugenia Belcastro PhD, Department of Translational Research and New Technologies in Medicine and Surgery, University of Pisa, 56126, Pisa, Italy.
Phone: +39 050 22 18555
Email: eugenia.belcastro@unipi.it

Table S1. Comparison between expression markers and different clinical-pathological features in pT1a and >pT1a melanoma groups.

Biomarker	Cellular subsets	Pathological features	n	Mean ± SD	*p*-value
		**TILs**			
β3-AR	Macrophages	No	3	15.33 ± 12.66	0.675
		Yes	11	20.11 ± 18.09	
	Melanocytes	No	3	33.33 ± 28.87	0.327
		Yes	11	55.91 ± 34.84	
HIF1- α	Lymphocytes	No	3	2.17 ± 2.47	0.748
		Yes	11	3.32 ± 5.78	
	Melanocytes	No	3	3.33 ± 5.77	0.426
		Yes	11	1.50 ± 2.71	
CD31		No	3	1.53 ± 0.93	0.973
		Yes	10	1.50 ± 1.58	
		**Histopathological ulceration**			
β3-AR	Macrophages	No	11	15.73 ± 13.16	0.151
		Yes	3	31.67 ± 25.66	
	Melanocytes	No	11	44.55 ± 34.31	0.178
		Yes	3	75.00 ± 22.91	
HIF1- α	Lymphocytes	No	11	2.91 ± 5.81	0.833
		Yes	3	3.37 ± 2.31	
	Melanocytes	No	11	0.73 ± 1.68	***0.007****
		Yes	3	6.17 ± 5.01	
CD31		No	10	1.14 ± 1.12	0.087
		Yes	3	2.73 ± 1.88	

Lesions with infiltrating lymphocytes (melanoma pT1a and melanoma >pT1a, n=11) compared to non-infiltrated melanocytic lesions (melanoma pT1a and melanoma >pT1a, n=3) and lesions with ulceration (melanoma pT1a and melanoma >pT1a, n=3) *versus* non-ulcerated lesions (melanoma pT1a and melanoma >pT1a, n=11). The continuous variables are summarized as mean ± standard deviation (SD). Values in bold italics indicate statistically significant results. Results were classified as statistically significant if their **p*-values were <0.05.

Table S2. Matrix of Pearson Correlation analysis

		**CD31**	**β3-AR**		**HIF1- α**		
			**Macrophages**	**Melanocytes**	**Lymphocytes**	**Melanocytes**	
**CD31**			0.737	0.465	-0.123	0.632	r
			<0.001*	***0.019****	0.557	***0.001****	*p*-value
			25	25	25	25	n
**β3-AR**	**Macrophages**	0.737		0.544	-0.108	0.63	r
		<0.001*		***0.003****	0.591	***<0.001****	*p*-value
		25		27	27	27	n
	**Melanocytes**	0.465	0.544		0.415	0.373	r
		0.019*	***0.003****		***0.032****	0.055	*p*-value
		25	27		27	27	n
**HIF1- α**	**Lymphocytes**	-0.123	-0.108	0.415		-0.004	r
		0.557	0.591	***0.032****		0.984	*p*-value
		25	27	27		27	n
	**Melanocytes**	0.632	0.63	0.373	-0.004		r
		0.001*	***<0.001****	0.055	0.984		*p*-value
		25	27	27	27		n

Biomarkers correlation analysis. * Correlation is significant at the 0.05 level (2-tailed).

Supplementary figure

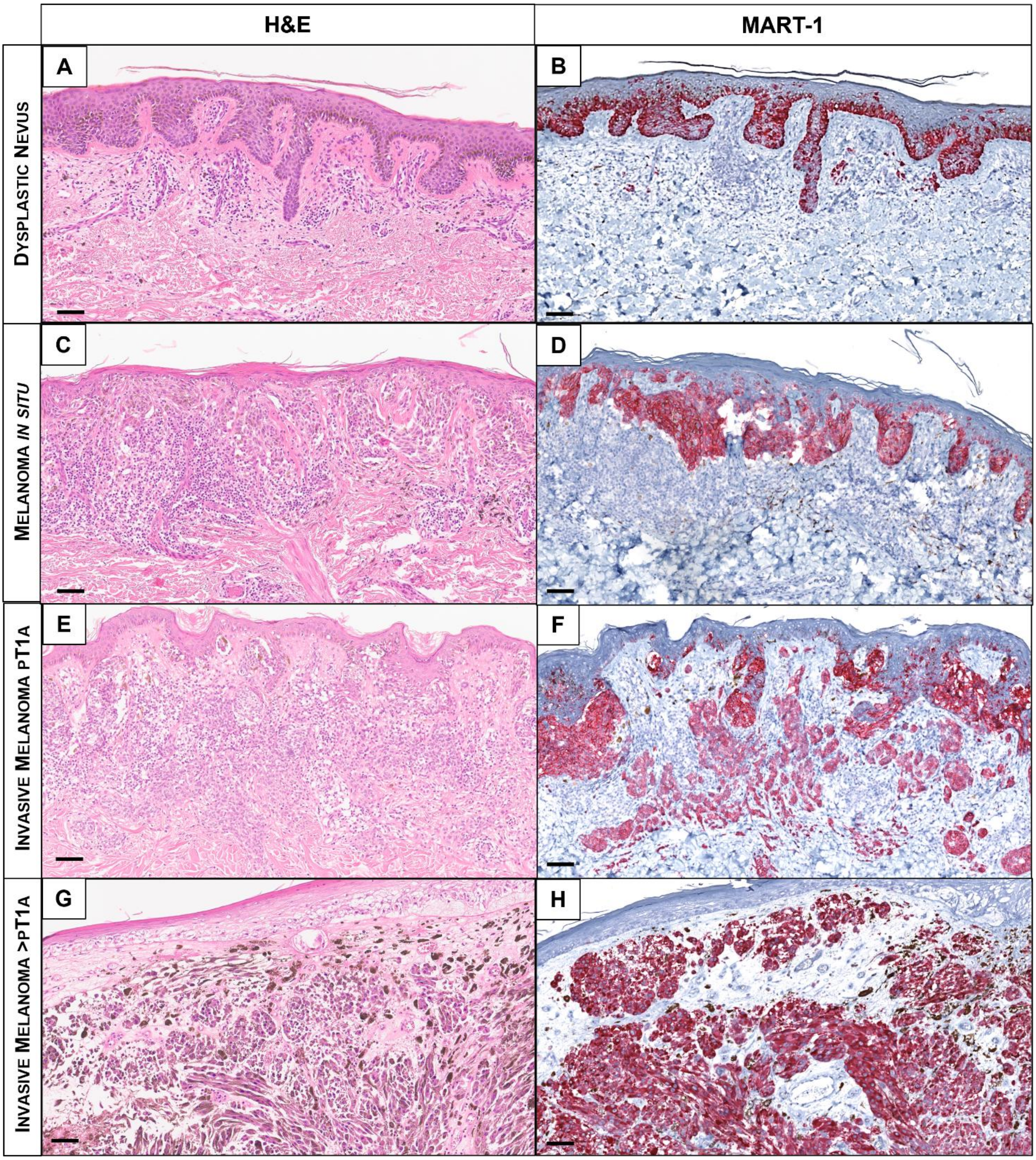

Figure S1. Representative images of H&E and MART-1 staining (in red) from the cases shown in Figure 2, (A, C, E, G) corresponding to the analysis of β3-AR expression in macrophages. Dysplastic nevus, H&E (**A**) and corresponding MART-1 staining (**B**), Melanoma in situ, H&E (**C**) and corresponding MART-1 staining (**D**), Melanoma pT1a, H&E (**E**) and corresponding MART-1 staining (**F**), and Melanoma >pT1a, H&E (**G**) and corresponding MART-1 staining (**H**). Please note that, for some samples, MART-1 staining was performed on

serial sections from the same FFPE block or on sections from additional FFPE blocks of the same lesion; therefore, the images may not strictly correspond to the exact sections shown in Figure 2.

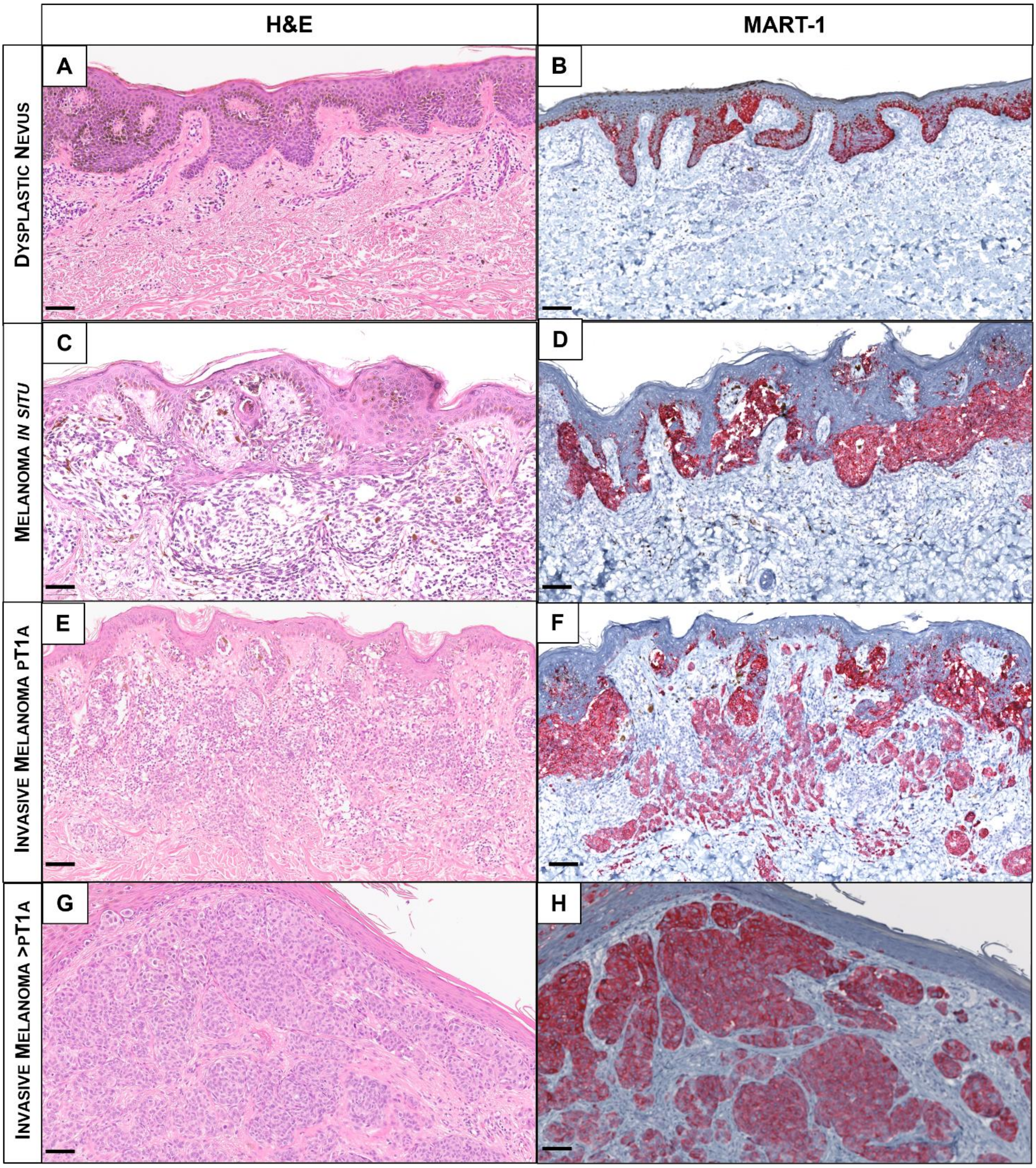

Figure S2. Representative images of H&E and MART-1 staining (in red) from the cases shown in Figure 2 (B, D, F, H) corresponding to the analysis of β3-AR expression in melanocytes. Dysplastic nevus, H&E (**A**) and corresponding MART-1 staining (**B**), Melanoma in situ, H&E (**C**) and corresponding MART-1 staining (**D**), Melanoma pT1a, H&E (**E**) and corresponding MART-1 staining (**F**), and Melanoma >pT1a, H&E (**G**) and

corresponding MART-1 staining (**H**). Please note that, for some samples, MART-1 staining was performed on serial sections from the same FFPE block or on sections from additional FFPE blocks of the same lesion; therefore, the images may not strictly correspond to the exact sections shown in Figure 2.

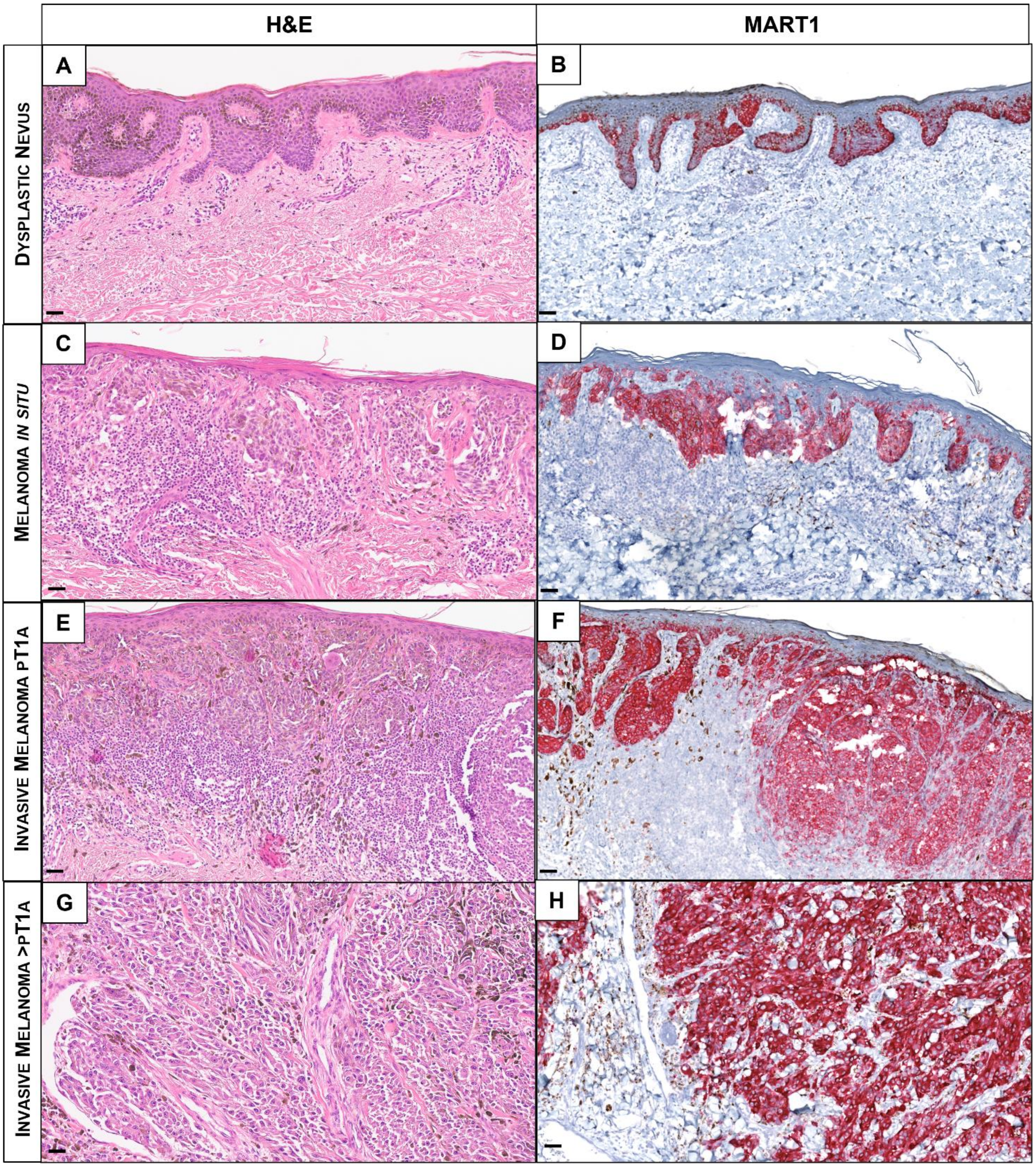

Figure S3. Representative images of H&E and MART-1 staining (in red) from the cases shown in Figure 3. Dysplastic nevi (**A, B**), Melanoma in situ (**C, D**), Melanoma pT1a (**E, F**), and Melanoma >pT1a (**G, H**). Please note that, for some samples, MART-1 staining was performed on serial sections from the same FFPE block or

on sections from additional FFPE blocks of the same lesion; therefore, the images may not strictly correspond to the exact sections shown in Figure 3.
